# The mitochondrial function of peripheral blood cells in cognitive frailty patients

**DOI:** 10.3389/fnagi.2024.1503246

**Published:** 2024-12-11

**Authors:** Li Qin, Tingting Huang, Danmei Zhang, Liqin Wei, Guochao Li, Qianqian Zhu, Qiangwei Tong, Guoxian Ding, Juan Liu

**Affiliations:** Division of Geriatric Endocrinology, The First Affiliated Hospital of Nanjing Medical University, Nanjing, China

**Keywords:** aging, cognitive frailty, mitochondrial function, peripheral blood cells, risk factors

## Abstract

**Background:**

Cognitive frailty (*CF*), characterized by the coexistence of physical frailty and cognitive impairment, is linked to increased morbidity and mortality in older adults. While *CF* has been linked to multiple physiological and lifestyle factors, the underlying biological mechanisms remain poorly understood. This study investigated the risk factors for *CF* and explored the relationship between mitochondrial function and *CF* in hospitalized patients.

**Methods:**

A total of 279 hospitalized individuals were recruited from December 2020 to August 2022, conducted comprehensive clinical assessments, and collected peripheral blood samples. *CF* was evaluated using the Physical Frailty Phenotype and Montreal Cognitive Assessment scales. Nutritional status was assessed with the Mini Nutritional Assessment, and depression was measured using the Geriatric Depression Scale. DNA was obtained from the peripheral blood and interrogated for mitochondrial DNA copy number (mtDNAcn). Peripheral blood mononuclear cells isolated from peripheral blood were examined for respiratory function and reactive oxygen species (ROS) levels. Additionally, plasma samples were analyzed for inflammatory markers and Carnitine Palmitoyltransferase II (CPT2).

**Results:**

Among the participants, 90 were classified as *CF* and 46 as non-*CF.* Logistic regression analysis revealed that increased age (OR 1.156, 95% CI 1.064–1.255), lower educational attainment (OR 0.115, 95% CI 0.024–0.550), malnutrition (OR 0.713, 95% CI 0.522–0.973), and higher depression scores (OR 1.345, 95% CI 1.065–1.699) were significantly associated with *CF.* The independent t tests and Mann–Whitney U tests showed the *CF* group exhibited impaired mitochondrial function, characterized by reduced mtDNAcn and respiratory activity, coupled with elevated ROS, interleukin-6, and CPT2 levels compared with the non-*CF* group. After adjusted for age, sex, and BMI, compared with non-*CF* group, the OR values for the *CF* group of mtDNAcn and ROS were 0.234 (95% CI = 0.065–0.849) (*p* = 0.027) and 1.203 (95% CI = 1.075–1.347) (*p* = 0.001), respectively. The Sensitive analysis showed that the area under curve values for mtDNAcn and ROS were 0.653 and 0.925.

**Conclusion:**

Age, lower educational attainment, malnutrition, and depression are significant risk factors for CF. Moreover, mitochondrial dysfunction, characterized by decreased mtDNAcn, impaired respiratory function and increased ROS levels appears to be a critical phenotype of *CF.*

## 1 Introduction

Since the early 21st century, the world has transitioned into an aging society, prioritizing healthy aging (Clegg et al., 2013; United Nations Department of Economic and Social Affairs, Population Division, 2024). And preserving cognitive function plays a crucial role in maintain health throughout the aging process (Davies et al., 2018). Generally, cognitive impairment is commonly recognized as a hallmark symptom in individuals with dementia, such as Alzheimer’s disease and vascular dementia. However, recent advancements in geriatric medicine have increasingly recognized cognitive frailty (*CF*), as a hidden yet pervasive syndrome, posing a significant threat to the cognitive health of many older adults. *CF*, introduced as a new concept in 2013, is characterized by the simultaneous presence of both physical frailty and mild cognitive impairment (MCI) (Panza et al., 2018; Kelaiditi et al., 2013; Sugimoto et al., 2022). While both *CF* and dementia in older adults involve cognitive impairment, they exhibit significant differences in clinical characteristics and pathological features. *CF* is defined as stable MCI that does not progress to dementia, whereas typical dementia is marked by a progressive decline in cognitive functions (Nader et al., 2023). Additionally, *CF* encompasses physical frailty, while dementia-related cognitive impairments tend to advance rapidly and are irreversible, with imaging studies revealing distinct changes such as brain atrophy and amyloid deposits (Kocagoncu et al., 2022).

Previous studies have shown that *CF* significantly increases risks for older adults, including falls, depression, malnutrition, disability, and higher hospitalization rates, positioning it as a predictor of adverse outcomes in this demographic (Guo X. et al., 2023; Zou et al., 2023; Tang et al., 2023; Qiu et al., 2023). Individuals with *CF* have a higher mortality rate compared with healthy older adults, those with MCI, and frail subjects, which places a substantial burden on society (Rivan et al., 2021; Vargas-Torres-Young et al., 2022). However, due to its potential reversibility, identifying risk factors and biomarkers for *CF*, coupled with targeted interventions, can facilitate healthier aging.

*CF* is reversible and may be mitigated through several interventions (Ibrahim et al., 2024; Hou et al., 2024; Tam et al., 2022; Li et al., 2022; Murukesu et al., 2020). Numbers of studies demonstrated that physical activity can ameliorate *CF.* A 24-month randomized controlled trial study reported that an organized moderate-intensity exercise can reduce *CF* (Liu et al., 2018). And another randomized controlled trial revealed that practicing mindfulness-based Tai Chi Chuan can boost both cognitive and physical functions in older adults (Jiayuan et al., 2022). Gutiérrez-Reguero et al. (2024) found that physical exercise can markedly enhance cognitive function, while dietary intervention alone cannot significantly improve cognitive frailty. Recently, Ibrahim et al. explored a multidimensional intervention for the reversal of *CF*, which consisted of vascular management, diet, exercise, cognitive and psychosocial stimulation (Ibrahim et al., 2024). Therefore, *CF* is not permanent and can potentially be alleviated through appropriate interventions, contributing to improved quality of life and independent living capabilities for older adults. And this feature of reversibility emphasizes the necessity for early detection and diagnosis of *CF.*

While *CF* has been linked to multiple physiological and lifestyle factors, the underlying biological mechanisms remain poorly understood. Mitochondria, known as the “energy factories” of the cells, are essential for supplying energy to neurons and regulating critical neuronal processes such as survival, regeneration, and the plasticity of axons and dendrites (Rangaraju et al., 2019). Impaired mitochondrial function in nerve cells is associated with cognitive decline, highlighting the close connection between mitochondrial health and cognitive abilities (Devine and Kittler, 2018; Apaijai et al., 2020). Restoration of mitochondrial function is critical for the management of cognitive dysfunction (Su et al., 2019). Previous studies reported that there was a negative correlation between mitochondrial DNA (mtDNA) levels in human peripheral blood mononuclear cells (PBMCs) and frailty, as well as cognitive decline (Ashar et al., 2015; Zhang et al., 2023a; Filograna et al., 2021; Tian et al., 2024). However, the relationship between mitochondrial function and *CF* remains unclear (Holland et al., 2024; Nader et al., 2023). Therefore, we hypothesize that mitochondrial dysfunction might be associated with *CF* and be a feature of *CF* in older adults.

This study aims to address this gap by investigating the risk factors associated with *CF* and exploring the relationship between mitochondrial function in peripheral blood and *CF* in hospitalized patients, offering insights into potential biomarkers and intervention strategies.

## 2 Methods

### 2.1 Participants, data collection and assessment scales

**Figure 1:**
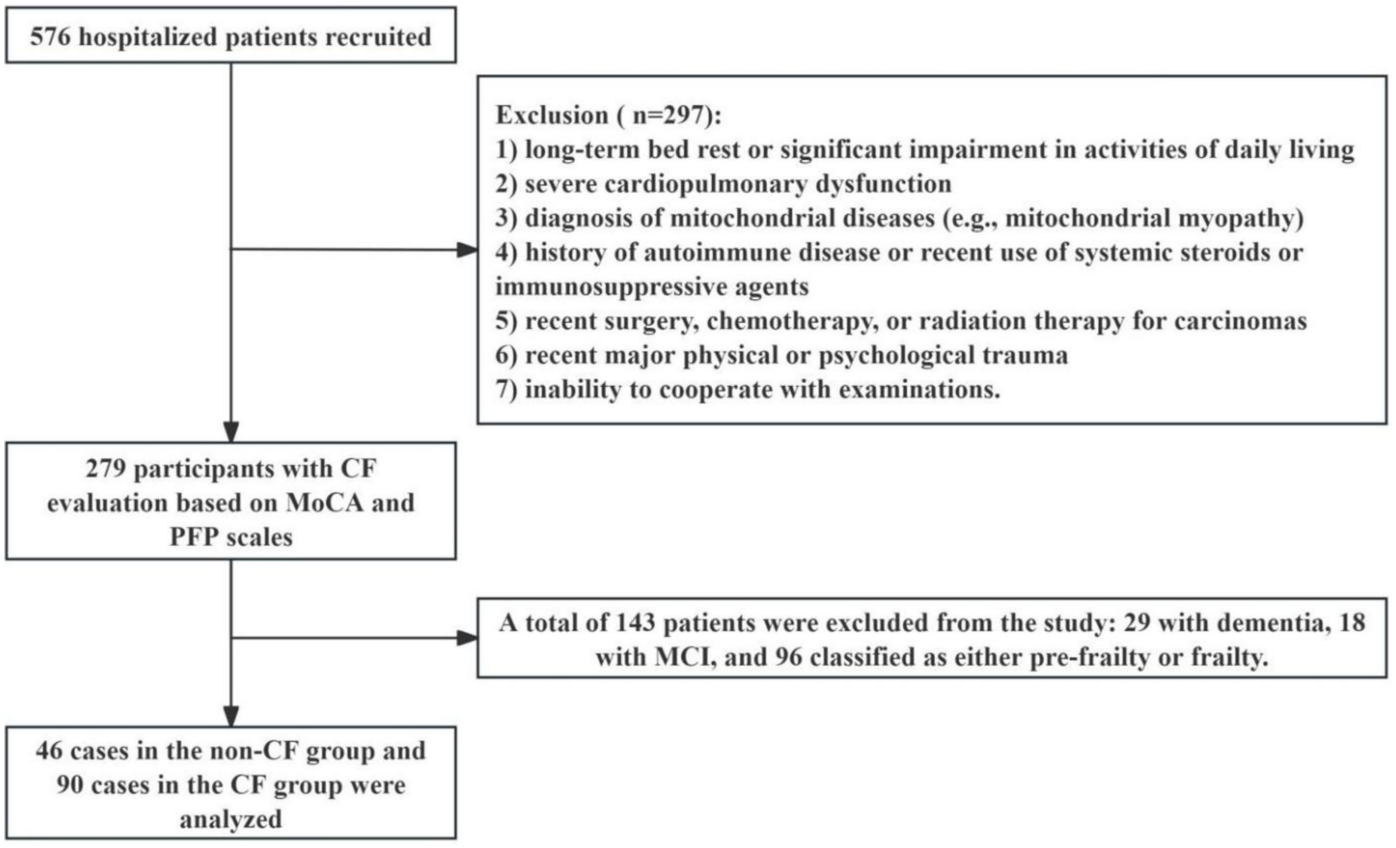
Flow chart of participants selection. *CF*: cognitive frailty, MCI, mild cognitive impairment; MoCA, the Montreal cognitive assessment; PFP, the physical frailty phenotype.

This study analyzed clinical data from 279 patients admitted to Jiangsu Province Hospital between October 2020 and August 2022 (Figure 1). Inclusion criteria were: (1) aged 50 years or older, (2) able to undergo all required examinations, and (3) stable underlying disease controlled by medication (for example, hypertension, coronary heart disease, diabetes, thyroid disease, etc.). Exclusion criteria included: (1) under 50 years of age, (2) long-term bed rest or significant impairment in activities of daily living, (3) severe cardiopulmonary dysfunction, (4) diagnosis of mitochondrial diseases (e.g., mitochondrial myopathy), (5) history of autoimmune disease or use of systemic steroids or immunosuppressive agents within the past 3 months, (6) recent (within 3 months) infection, surgery, chemotherapy, or radiation therapy for carcinomas, (7) recent (within 3 months) major physical trauma or diagnosed with depression, which means psychological impact of distressing events, major including accidents and natural disasters within last 3 months; and (8) inability to cooperate with examinations. The Ethical Committee of Jiangsu Province Hospital approved the study (2019-NT-48, 2024-SR-087), and all participants provided signed informed consent.

Patients underwent assessments across various health dimensions: (1) *CF* status measured with the Montreal Cognitive Assessment (MoCA) and the Physical Frailty Phenotype (PFP). Frailty status was assessed based on PFP including five components (Fried et al., 2001): PFP score < 1 = non-frail, 1 ≤ PFP score < 3 = pre-frail, 3 ≤ PFP score ≤ 5 = frail; Cognitive function was evaluated based on MoCA (Nasreddine et al., 2005): 26 ≤ MoCA score = normal cognitive function, 15 ≤ MoCA score < 26 = mild cognitive impairment (MCI), MoCA score ≤ 15 = dementia. Subjects were classified into the non-*CF* group if they had a PFP score = 0 and a MoCA score ≥ 26. In contrast, subjects with a PFP score of ≥1 and a MoCA score between 16 and 25 were classified into the *CF* group; (2) Mental Health, measured with the Self-Rating Anxiety Scale (SAS) and the Geriatric Depression Scale (GDS). SAS: total score ranges from 20 to 80, and higher scores correspond to a more pronounced tendency toward anxiety. GDS: total score ranges from 0 to 30, and higher scores indicate greater severity of depressive symptoms. (3) Physical Function, assessed with the Timed Up and Go test (TUG), the Short Physical Performance Battery (SPPB), and the strength, assistance walking, rise from a chair, climb stairs, and falls combined with calf circumference (SARC-Calf); (4) Nutritional Status, determined via the Mini Nutritional Assessment (MNA); (5) Fall Risk, evaluated using the Morse Fall Scale (MFS); (6) Comorbidity, assessed using the Cumulative Illness Rating Scale - Comorbidity Index (CIRS-CI).

### 2.2 Body composition

Dual-energy X-ray absorptiometry (DXA) (HOLOGIC, US) scans provided detailed assessments of body composition, including total fat mass, android and gynoid fat mass, and appendicular skeletal muscle mass. These measurements facilitated the calculation of the Fat Mass Index (FMI, defined as total fat mass divided by height squared), the Android-to-Gynoid (A/G) ratio, and the Appendicular Skeletal Muscle Mass Index (ASMI, calculated as appendicular skeletal muscle mass divided by height squared).

### 2.3 Total DNA extracted from peripheral blood

A total of 200 μL of fresh human venous blood was collected from participants using Vacutainer tubes containing sodium heparin as an anticoagulant. Total DNA was extracted from this peripheral blood, yielding a final volume of 75 μL, utilizing the FastPure^®^ Blood DNA Isolation Mini Kit V2 (Vazyme, China), according to the manufacturer’s instructions.

### 2.4 Quantitative real-time polymerase chain reactions (qRT-PCR)

Mitochondrial DNA copy number (mtDNAcn) was assessed using Quantitative Real-Time Polymerase Chain Reactions (qRT-PCR) Instrument (Thermo Fisher, US). The reactions were conducted in a 10 μL volume, consisting of 2 μL of mtDNA template and 5 μL of ChamQ Universal SYBR qPCR Master Mix (Vazyme, China). qRT-PCR data were analyzed using the 2 − ΔΔCT method. The forward primer sequence for *β*-actin: ATTGGCAATGAGCGGTTCCGC, reverse primer: CTCCTGCTTGCTGATCCACATC; Forward primer sequence for MT-ND-1: CACTCACATCACAGCGCTAA; reverse primer: GGATTATGGATGCGGTTGCT.

### 2.5 Isolation of peripheral blood mononuclear cells (PBMCs)

All blood samples were obtained after overnight fasting. Two milliliters of freshly collected whole blood, anticoagulated with heparin, were obtained from participants for the isolation of PBMCs (T cells, B cells, dendritic cells, monocyte, phagocyte, natural killer cells, and a few other cell types) and plasma via Ficoll gradient centrifugation (TBD, China), in accordance with the manufacturer’s guidelines. The isolated PBMCs were subsequently used to evaluate oxygen consumption rate (OCR) and reactive oxygen species (ROS), while the plasma was stored at −80°C Refrigerator (Sanyo, Japan) for later analysis.

### 2.6 Oxygen consumption rate measurements (OCR) in mitochondria of PBMCs

OCR measurements in PBMCs were conducted using a Seahorse XFe24 extracellular flux analyzer (Agilent, US), following the manufacturer’s instructions. Briefly, 2 × 10^5^ PBMCs were seeded onto Cell-Tak-coated XF24 V7 PS cell culture microplates in XF assay medium and incubated at 37°C for 1 h in a non-CO2 incubator. The XF assay medium was comprised of XF DMEM medium supplemented with 1 mM pyruvate, 1 mM glutamine, and 1 mM glucose. Subsequently, OCR was monitored at baseline and during sequential injections of oligomycin (1.5 μM), Carbonyl cyanide-4 (trifluoromethoxy) phenylhydrazone (FCCP, 0.5 μM), and a mix of rotenone and antimycin A (Rot/AA, 0.5 μM for each), using the XFe24 system. Data were collected and analyzed with Wave software and Microsoft Excel. The following mitochondrial respiration parameters were calculated: Basal Respiration = (baseline OCR) **-** (OCR after rotenone/antimycin A treatment), ATP-Linked Respiration = (Basal Respiration) **-** (OCR after Oligomycin treatment), Proton leak = (OCR after Oligomycin treatment) **-** (OCR after rotenone/antimycin A treatment), Maximal Respiration = (OCR after FCCP treatment) **-** (OCR after rotenone/antimycin A treatment), Spare Respiratory Capacity = (Maximal Respiration) **-** (Basal Respiration).

### 2.7 Reactive oxygen species (ROS) detection in PBMCs and flow Cytometric analysis

ROS levels in PBMCs were assessed using a Reactive Oxygen Species Assay Kit (Beyotime, China), according to the manufacturer’s instructions. Notably, 2′,7′-Dichlorofluorescin diacetate (DCFH-DA) is a non-fluorescent probe that can be oxidized by reactive oxygen species (ROS), resulting in the formation of the highly fluorescent dichlorofluorescein (DCF). Freshly isolated PBMCs were incubated with 10 μmol/L DCFH-DA probes at 37°C for 20 min, with mixing every 3 min. PBMCs were then washed three times with phosphate-buffered saline (PBS). Sample analysis was performed using flow cytometer (BD, USA).

### 2.8 Enzyme-linked immunosorbent assay (ELISA)

Concentrations of inflammatory markers (IL-1β, IL-6, TNF-*α*) (Multi Sciences, China) and metabolites (CPT2, L-Carnitine) (Maisha, China) in plasma were measured using ELISA kits, following the manufacturers’ protocols (Niu et al., 2024; Xuekelati et al., 2024). Optical density was measured at wavelengths of 570 nm and 630 nm using a microplate reader.

### 2.9 Statistical analysis

Data were analyzed using IBM SPSS Statistics Version 25. Normality was assessed using measures of Skewness and Kurtosis, the Shapiro–Wilk test, histograms, and Q-Q plots. Continuous variables were expressed as mean ± standard deviation (SD). We first examined differences in participants’ characteristics by *CF* status. We used independent t tests or Mann–Whitney U tests for used independent t tests for continuous variables, and chi-square for categorical variables, as appropriate. To investigate the risk factors of *CF*, we used the binary logistic regression models, including the univariate and multivariate analyses, which adjusted for age, sex, BMI, education level, (MNA) scores, and (GDS) scores. We then examined the association between the level of mtDNAcn, mitochondrial respiration, ROS, CPT2, L-Carnitine, IL-1β, IL-6, or TNF-*α* and *CF* using the independent t tests or Mann–Whitney U tests, as appropriate. To adjust for age, sex, and BMI, we also used logistic regression analysis to investigate the relationship between mtDNAcn or ROS and *CF.* Furthermore, to evaluate whether mtDNAcn or ROS may be a predictor of *CF*, we used the sensitivity analysis to analyze the sensitivity of data. Differences in mitochondrial function between groups were illustrated through columnar scatter plots generated using GraphPad Prism software, and ROS levels were analyzed with FlowJo software. The sensitivity analysis was conducted by IBM SPSS Statistics Version 25 and GraphPad Prism software. Statistical significance was defined at *p* < 0.05.

## 3 Results

### 3.1 Demographics and group distribution

This study involved 279 participants, with a mean age of 73.63 (± 10.19 years) and a Body Mass Index (BMI) of 23.95 (± 3.21 kg/m^2^), including 185 males (66.31%) and 94 females (33.69%). Participants were categorized based on the PFP and the MoCA scales. The non-*CF* group (*n* = 46) exhibited no signs of frailty and maintained normal cognitive function, while the *CF* group (*n* = 90) showed indicators of pre-frailty or frailty combined with MCI. An additional 143 participants were identified as having conditions such as frailty, MCI, or dementia.

Comorbidities included hypertension (47.83% in the non-*CF* group, 22 cases; 32.72% in the *CF* group, 53 cases), diabetes (39.13% in the non-*CF* group, 18 cases; 25.17% in the *CF* group, 37 cases), coronary heart disease (10.87% in the non-*CF* group, 5 cases; 35.94% in the *CF* group, 23 cases), malnutrition (4.35% in the non-*CF* group, 2 cases; 44.83% in the *CF* group, 26 cases), and dyslipidemia (43.48% in the non-*CF* group, 20 cases; 31.25% in the *CF* group, 35 cases). The hospital length of stay was 8.98 ± 1.20 days for the non-*CF* group and 12.90 ± 0.94 days for the *CF* group. There were notable differences in malnutrition and the hospital length of stay between the two groups (Table 1). Additionally, in the assessment of *CF* using the MoCA and PFP scales in 279 participants, 143 individuals were excluded, including 29 diagnosed with dementia, 18 with MCI without frailty, and 96 classified as pre-frail or frail without MCI.

**Table 1:** The overall characteristics of participants.

Variables	non-CF group (*n* = 46)	CF group (*n* = 90)	*p* value
Age (year)	66.15 ± 0.92	78.51 ± 1.03	<0.0001
Sex (F), *n* (%)	9 (19.57)	27 (30.00)	0.19
Education, *n* (%)			
Illiterate	0 (0)	2 (2.22)	0.0001
Primary	0 (0)	10 (11.11)	
Secondary	4 (8.70)	32 (35.56)	
College	42 (91.30)	46 (51.11)	
Smoking (yes)	12 (26.09)	58 (64.44)	0.32
Drinking (yes)	22 (47.83)	29 (32.22)	0.06
Hypertension, n (%)	22 (47.83)	53 (32.72)	0.27
Diabetes, n (%)	18 (39.13)	37 (25.17)	0.82
CHD, n (%)	5 (10.87)	23 (35.94)	0.05
Malnutrition, n (%)	2 (4.35)	26 (44.83)	0.001
Dyslipidemias, n (%)	20 (43.48)	35 (31.25)	0.61
LOS (day)	8.98 ± 1.20	12.90 ± 0.94	0.001
BMI (kg/m^2^)	25.37 ± 0.47	23.46 ± 0.31	0.001
WHR	0.96 ± 0.01	0.94 ± 0.01	0.27
FMI (kg/m^2^)	8.18 ± 0.37	8.11 ± 0.32	0.89
ASMI (kg/m^2^)	6.51 ± 0. 19	5.62 ± 0.14	<0.0001
A/G	0.72 ± 0.03	0.65 ± 0.02	0.02
PFP scores	0	2.22 ± 0.12	<0.0001
MoCA scores	28.31 ± 0.21	21.53 ± 0.33	<0.0001
MFS scores	17.80 ± 1.24	30.75 ± 2.00	<0.0001
SPPB scores	11.62 ± 0.13	9.01 ± 0.76	<0.0001
Strength grip (kg)	34.74 ± 1.17	25.44 ± 0.95	<0.0001
4 meters gait speed (m/s)	1.39 ± 0.05	1.02 ± 0.04	<0.0001
TUG-single(s)	6.64 ± 0.18	9.38 ± 0.30	<0.0001
TUG-man(s)	9.34 ± 0.25	13.18 ± 0.43	<0.0001
TUG-cog(s)	9.24 ± 0.34	13.53 ± 0.51	<0.0001
SARC-calf	2.95 ± 1.00	6.73 ± 0.89	0.003
MNA scores	27.42 ± 0.37	24.66 ± 0.47	<0.0001
GDS scores	2.95 ± 0.35	8.47 ± 0.59	<0.0001
SAS scores	23.72 ± 0.78	27.55 ± 0.76	0.002
CIRS-CI scores	1.72 ± 0.23	3.09 ± 0.21	<0.0001
HbA1c (%)	6.23 ± 0.16	6.33 ± 0.11	0.32
GLU (mmol/L)	5.28 ± 0.22	5.36 ± 0.13	0.41
INS (pmol/L)	84.15 ± 10.66	48.44 ± 4.42	0.002
TC (mmol/L)	4.26 ± 0.15	4.18 ± 0.11	0.69
TG (mmol/L)	1.40 ± 0.09	1.23 ± 0.06	0.01
HDL (mmol/L)	1.10 ± 0.04	1.13 ± 0.03	0.63
LDL (mmol/L)	2.56 ± 0.11	2.49 ± 0.08	0.60
TP (g/L)	64.08 ± 0.86	63.47 ± 0.55	0.55
ALB (g/L)	39.05 ± 0.45	37.57 ± 0.38	0.02
RBP (mg/L)	41.06 ± 1.50	37.16 ± 1.07	0.04
PA (g/L)	0.28 ± 0.01	0.23 ± 0.01	<0.0001
TRF (g/L)	2.14 ± 0.06	2.01 ± 0.05	0.12
HGB (g/L)	138.10 ± 2.19	117.29 ± 3.66	0.001
VIT-D (nmol/L)	65.05 ± 3.05	61.97 ± 4.89	0.79
FT3 (pmol/L)	4.54 ± 0.083	4.15 ± 0.080	0.003
FT4 (pmol/L)	16.39 ± 0.38	16.27 ± 0.26	0.80
TSH (mIU/L)	2.93 ± 0.28	2.39 ± 0.16	0.32

Categorical data were presented as frequencies and percentages, and continuous variables were expressed as mean ± standard deviation (SD). Differences between groups were evaluated using Chi-square test, Student’s *t*-tests, or Mann–Whitney U tests, as appropriate. Statistical significance was defined at *P* < 0.05. CF, cognitive frailty; CHD, coronary atherosclerotic heart disease; LOS, length of stay; BMI, body mass index; WHR, waist-to-hip ratio; FMI, fat mass index; ASMI, appendicular skeletal Muscle Mass Index; A/G, Android-to-Gynoid ratio; PFP, physical frailty phenotype; MoCA, Montreal cognitive assessment; MFS, Morse fall scale; SPPB, short physical performance battery; TUG, timed up and go test; SARC-calf, strength, assistance walking, rise from a chair, climb stairs, and falls combined with calf circumference; MNA, mini nutritional assessment; GDS, geriatric depression scale; SAS, self-rating anxiety scale; CIRS-CI, cumulative illness rating scale - comorbidity index; HbA1c, Glycosylated Hemoglobin; GLU, glucose; INS, insulin; TC, total cholesterol; TG, triglyceride; HDL, high-density lipoprotein; LDL, low-density lipoprotein; TP, total protein; ALB, albumin; RBP, retinal binding protein; PA, prealbumin; TRF, transferrin; HGB, hemoglobin; VIT-D, 25-hydroxyvitamin D; FT3, free triiodothyronine; FT4, free thyroxine; TSH, thyrotrophin.

### 3.2 Clinical factors and group comparisons

Baseline characteristics are summarized in Table 1. The *CF* group was significantly older and had higher scores on the MFS, TUG, GDS, SAS, and CIRS-CI. Conversely, the *CF* group exhibited significantly lower educational attainment, BMI, ASMI, A/G ratios, SPPB scores, handgrip strength, 4-meter gait speed, and MNA scores, and various biochemical markers, including insulin, triglycerides, albumin, retinol-binding protein, prealbumin, hemoglobin, and free triiodothyronine. No significant differences were found in gender distribution, waist-to-hip ratio (WHR), smoking or drinking history, FMI, Glycosylated Hemoglobin, total cholesterol, glucose, low-density lipoprotein, high-density lipoprotein, total protein, transferrin, free thyroxine, thyroid-stimulating hormone, and vitamin D between the groups.

**Figure 2:**
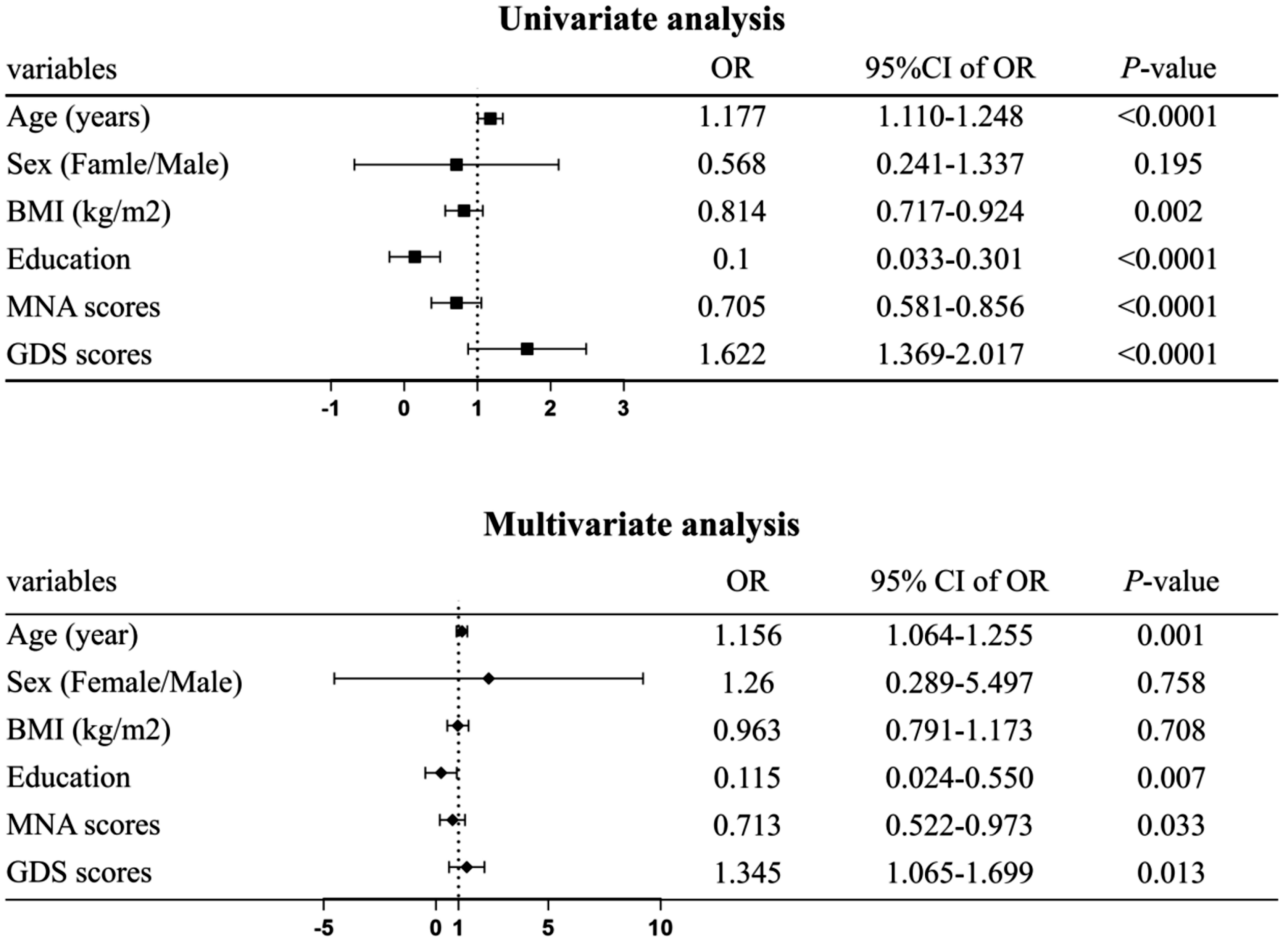
Forest plot factors significantly associated with cognitive frailty (*CF*). Data were analyzed using binary logistic regression. Adjusted for age, sex, body mass index (BMI), education level, mini nutritional assessment (MNA) scores, and geriatric depression scale (GDS) scores. Statistical significance was defined at *p* < 0.05.

The binary logistic regression model included sex, age, BMI, education, MNA scores, and GDS scores (Figure 2). Results showed that older age was associated with increased of *CF* risk (OR 1.156, 95% CI 1.064–1.255), lower educational levels linked to higher *CF* risk (OR 0.115, 95% CI 0.024–0.550), declining MNA scores indicated greater *CF* risk (OR 0.713, 95% CI 0.522–0.973), and higher GDS scores correlated with increased *CF* risk (OR 1.345, 95% CI 1.065–1.699).

### 3.3 Correlation between mitochondrial function and *CF*

**Figure 3:**
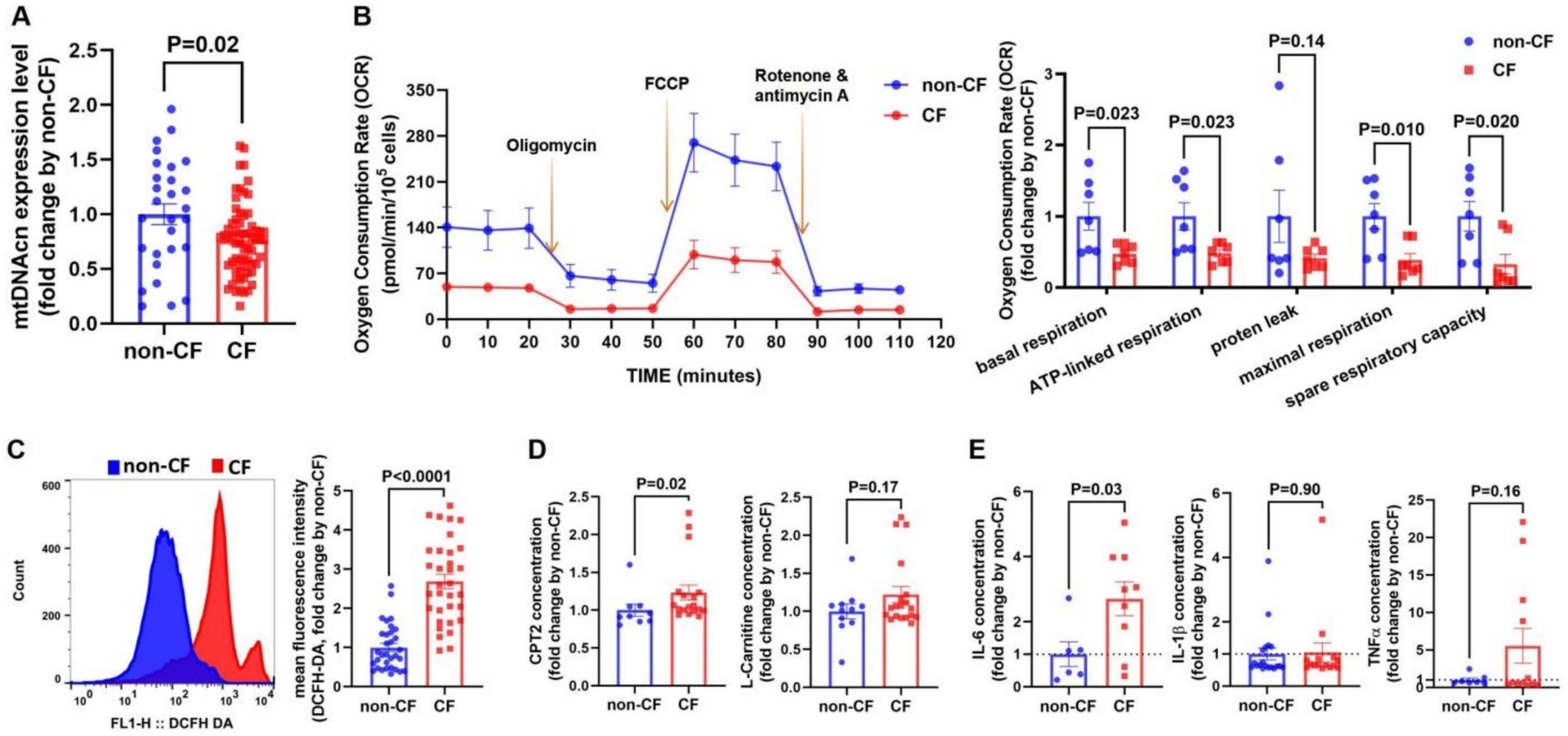
Mitochondrial dysfunction and inflammatory cytokines levels are increased in the cognitive frailty (*CF*) group. **(A)** qPCR was used to validate mitochondrial DNA copy number (mtDNAcn) levels in peripheral blood n (non-*CF*) = 28, n (*CF*) = 64. **(B)** Peripheral blood mononuclear cells (PBMCs) PBMCs underwent a mitochondrial stress test to measure oxygen consumption rate (OCR). Arrows indicate the addition of oligomycin, carbonyl cyanide-4 (trifluoromethoxy) phenylhydrazone (FCCP), and rotenone/antimycin A (left). Parameters analyzed included basal espiration, ATP-linked respiration, proten leak, maximal respiration, and spare respiratory capacity (right). n (non-*CF*) = 7, n (*CF*) = 7. **(C)** Flow cytometry analysis of DCFH-DA expression in PBMCs (left). Quantitative fluorescence analysis of DCFH-DA (right). n (non-*CF*) = 32, n (*CF*) = 33. **(D)** Secretion levels carnitine palmitoyltransferase 2 (CPT2) [n (non-*CF*) = 9, n (*CF*) = 18], and L-carnitine [n (non-*CF*) = 11, n (*CF*) = 19]. **(E)** Cytokines levels of interleukin-6 (IL-6) [n (non-*CF*) = 6, n (*CF*) = 9], interleukin-1 beta (IL-1β) [n (non-*CF*) = 7, n (*CF*) = 12] and tumor necrosis factor-α (TNF-α) [n (non-*CF*) = 19, n (*CF*) = 15] in plasma determined by ELISA. Notes: Differences in mitochondrial function between the two groups were illustrated using columnar scatter plots generated with GraphPad Prism software, while ROS levels were analyzed with FlowJo. Statistical comparisons were evaluated using Mann–Whitney U tests or Student’s *t*-tests, as appropriate. Data are presented as means ± standard error of the mean (SEM). Statistical significance was defined at *p* < 0.05.

Our results indicated that the *CF* group had lower mtDNAcn expression levels compared with the non-*CF* group (Figure 3A). Additionally, key mitochondrial respiratory function parameters—such as basal respiration, ATP-linked respiration, maximal respiration, and spare respiratory capacity—were significantly reduced in the *CF* group, while proton leak did not show significant differences between the groups (Figure 3B). ROS levels were higher in the *CF* group relative to the non-*CF* group (Figure 3C). Notably, although plasma levels of Carnitine Palmitoyltransferase II (CPT2) were elevated in the *CF* group, circulating levels of L-carnitine did not differ statistically (Figure 3D).

After adjustment for age, sex, and BMI, compared with non-*CF* group, the OR values for the *CF* group of mtDNAcn and ROS were 0.234 (95% CI = 0.065–0.849) (*p* = 0.027) and 1.203 (95% CI = 1.075–1.347) (*p* = 0.001), respectively. Similar results were obtained in both the unadjusted and the age-sex-adjusted model (Table 2).

**Table 2:** Binary logistic regression model.

Variables	OR (95% CI)	*p* **-value**	OR (95% CI)	*P* **-value**
	Model 1		Model 2	
mtDNAcn	0.299 (0.116–0.773)	0.013	0.310 (0.098–0.983)	0.047
ROS	1.156 (1.078–1.240)	<0.0001	1.281 (1.087–1.365)	0.001
	Model 3		Model 4	
mtDNAcn	0.248 (0.069–0.891)	0.033	0.234 (0.065–0.849)	0.027
ROS	1.205 (1.080–1.344)	0.001	1.203 (1.075–1.347)	0.001

Data were analyzed using binary logistic regression. Model 1: Crude model; Model 2: adjust for age; Model 3: adjust for age, sex; Model 4: adjust for age, sex, BMI. Statistical significance was defined at *P* < 0.05. mtDNAcn: Mitochondrial DNA copy number; ROS: Reactive Oxygen Species; OR: odds ratio; CI: confidence interval.

### 3.4 Correlation between inflammatory cytokines levels and *CF*

In addition, plasma IL-6 levels were elevated in the *CF* group compared with the non-*CF* group, while no significant differences were found for IL-1β or TNF-*α* levels between the groups (Figure 3E).

### 3.5 The diagnostic value of mtDNAcn and ROS for *CF*

**Figure 4:**
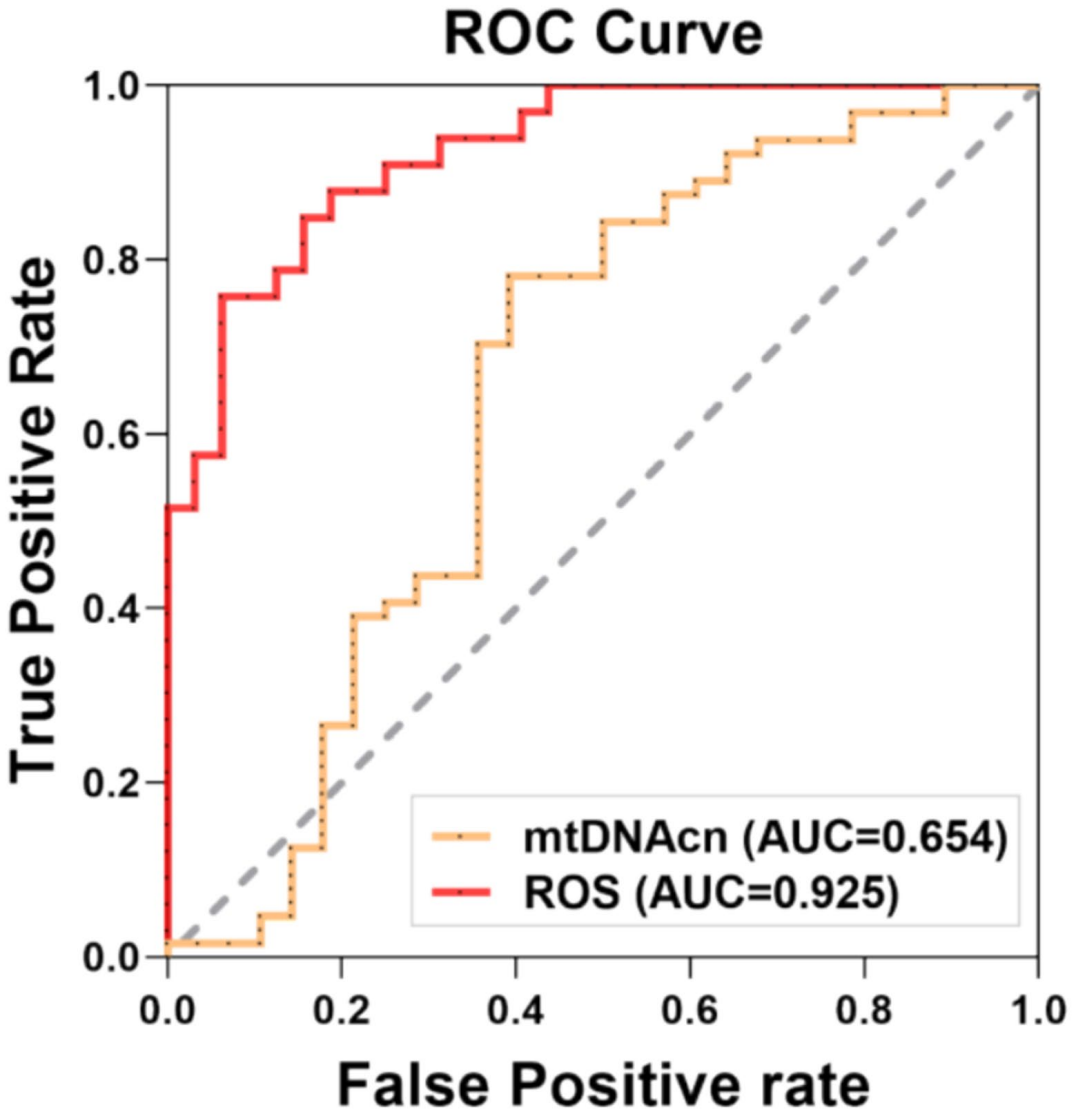
Receiver operating characteristic (ROC) curve for the prediction model. The sensitivity analysis was conducted by ROC curve, which was assessed the capacity of mtDNAcn and ROS to predictive *CF*, respectively. Statistical significance was defined at *p* < 0.05. AUC: area under the curve, mtDNAcn: mitochondrial DNA copy number, ROS: reactive oxygen species.

The *CF* group was designated as the positive cohort, while the non-*CF* group served as the negative cohort for plotting the ROC curve. The analysis revealed that the area under curve (AUC) values for mtDNAcn and ROS in predicting *CF* were 0.653 and 0.925, respectively, as shown in Table 3 and Figure 4.

**Table 3:** The diagnostic value of mtDNAcn and ROS for cognitive frailty.

Variables	AUC	95% CI	*P*	Cut-Off	Sensitivity	Specificity
mtDNAcn	0.653	0.515–0.792	0.019	< 0.953	0.781	0.607
ROS	0.925	0.866–0.985	<0.001	> 321	0.758	0.937

The sensitivity analysis was used to analyze the effective predictor of CF. Statistical significance was defined at *P* < 0.05. mtDNAcn, mitochondrial DNA copy number; ROS, reactive oxygen species. AUC, area under the curve; CI, confidence interval.

## 4 Discussion

This study identified a 32.26% prevalence of *CF* among inpatients. Our results showed that the *CF* group had a longer hospital length of stay compared with the non-*CF* group. The independent t tests, Mann–Whitney U tests and binary logistic regression suggested that notable risk factors of *CF* included advancing age, lower educational attainment, malnutrition, and depressive states. The study included 9 females (19%) in the non-*CF* group and 27 females (30%) in the *CF* group. While women were generally more frail, they appeared less susceptible to mortality than men (Arosio et al., 2023; Bai et al., 2023). To assess potential gender differences between the two groups, we performed a chi-square test (χ^2^ = 1.703, *p* = 0.192) and a multivariate logistic regression analysis (95% CI: 0.289–5.497, *p* = 0.758), both of which indicated no significant effect of gender on *CF.* We found that lower levels of insulin, triglycerides, albumin, retinol-binding protein, prealbumin, hemoglobin, and free triiodothyronine in the *CF* group. Sugimoto et al. also reported the risk factors of *CF* included sex, age, education level, socioeconomic status, malnutrition, and depressive states (Sugimoto et al., 2022). Moreover, multidomain lifestyle interventions for the older people, which incorporating exercise, nutritional support, and cognitive training, have proven effective in enhancing both physical frailty and cognitive function (Murukesu et al., 2020; Zhang et al., 2023b). Therefore, early detection, diagnosis, and intervention are essential for managing the onset and progression of *CF.*

Previous studies have utilized human peripheral blood samples as biomarkers for diagnosing various conditions, including heart failure, cognitive impairments, frailty, and dementia (Alexovič et al., 2022; Mahapatra et al., 2023; Lee et al., 2022). Furthermore, human PBMCs are widely recognized as critical targets for assessing mitochondrial function (Silaidos et al., 2018). *CF*, characterized as a geriatric syndrome affecting both physical and cognitive functions, is linked not only to mitochondrial dysfunction in specific tissues but also to systemic aging of tissues and organs (Robinson et al., 2022; Ruan et al., 2017; Zhang X. M. et al., 2022; Arosio et al., 2023; Ma and Chan, 2020). Consequently, to analyze the correlation between *CF* and mitochondrial function, we chose to assess mitochondrial function in peripheral blood cells rather than focusing on specific tissues such as skeletal muscle.

MtDNAcn quantifies mitochondrial content, influenced by various factors. Previous studies have linked elevated mtDNAcn levels in whole blood to an increased risk of glioma carcinogenesis, while lower mtDNAcn has been associated with greater susceptibility to aging and age-related diseases, such as coronary heart disease, cognitive impairments, and frailty (Shen et al., 2016; Wachsmuth et al., 2016; Mengel-From et al., 2014; Ashar et al., 2015; Klein et al., 2021; Yang et al., 2021). A review on aging and mtDNA suggested that most studies reported a decrease in mtDNAcn with aging (Filograna et al., 2021). Tian et al. demonstrated that higher mtDNAcn in human blood is associated with lower cognitive decline and frailty risk (Tian et al., 2024). However, a study reported that mtDNAcn increased with aging in liver samples (Wachsmuth et al., 2016). And another study showed no correlation between age and mitochondrial health index in PBMC (Karan et al., 2020). The variability in the relationship between age and mtDNAcn may be attributed to the heterogeneous composition of leukocytes and platelet abundance, which fluctuate with time of day, aging, and disease (Picard, 2021). In this study, the results demonstrated that the mtDNAcn in the *CF* group was lower than that in the non-*CF* group. After adjusted for age, sex, and BMI, our results still suggested that reduced mtDNAcn remained positively correlated with *CF.* Moreover, the Sensitivity analysis suggested that mtDNAcn could be a key parameter for evaluating *CF.* Therefore, low mtDNAcn level in blood is associated with *CF* and may serve as a characteristic feature of *CF.*

Mitochondrial respiration is essential for maintaining normal cellular function. Mitochondrial health is particularly crucial in age-related disorders, including cardiovascular and neurodegenerative diseases, which often stem from imbalances in energy supply and demand (Amorim et al., 2022). The brain, being a high-energy-demand organ, is especially vulnerable to deficits in oxygen and energy. Mitochondrial dysfunction can trigger pathological responses in neural tissues, leading to cognitive decline (Yin et al., 2016; Cunnane et al., 2020). Moreover, Mahapatra et al., 2023 confirmed a positive association between mitochondrial respiratory capacity in human PBMCs and both cognitive ability and hippocampal volume. Additionally, aging-related impairments in object recognition and spatial memory have been linked to reduced synaptic mitochondrial ATP production in the hippocampus (Olesen et al., 2020). Ultimately, a decrease in neural energy availability may contribute to cognitive decline through impaired neural structure and function (Cheng et al., 2022; Rangaraju et al., 2019). Our study indicated that the *CF* group exhibited lower basal respiration, ATP-linked respiration, maximal respiration, and spare respiratory capacity compared with the non-*CF* group. These findings suggest that impaired mitochondrial respiration is associated with *CF.*

Mitochondria are the primary intracellular source of ROS; and previous reviews have suggested a potential link between *CF* and increased oxidative stress due to mitochondrial dysfunction (Bertero and Maack, 2018; Zhang B. et al., 2022). While ROS generated by the mitochondrial respiratory chain are critical signaling molecules in healthy cells, their excessive production during oxidative stress can lead to mtDNA damage, mitochondrial dysfunction, and cell death (Zarse and Ristow, 2021). In general, age-related mitochondrial abnormalities, such as accumulation of mtDNA mutations, diminished respiratory chain activity, and increased ROS generation, are implicated in the aging process. Some reviews proposed that the balance between ROS generation and clearance were disturbed in aging and neurodegenerative diseases (Guo Y. et al., 2023; Ionescu-Tucker and Cotman, 2021). And in aging animal models, ROS production in synaptic mitochondria disrupts essential neuronal proteins, contributing to cognitive decline (Kron et al., 2020; Olesen et al., 2020). Our results showed that ROS levels in PBMCs were significantly higher in the *CF* group compared with that in the non-*CF* group. After adjusted for age and sex, we observed that ROS levels in the *CF* group were significantly higher than that in the non-*CF* group. Furthermore, our results demonstrated that ROS levels might be an important parameter to assess *CF.* Therefore, high levels of ROS is correlated with *CF*, and may serve as a critical feature of *CF.*

Cellular stress can alter membrane permeability, and mitochondrial damage and its byproducts may exacerbate inflammation (Picca et al., 2021; Vringer and Tait, 2023). Recent studies suggested that mitochondria modulate inflammation through signaling pathways involving mtDNA and ROS, which trigger the release of TNF-*α* and IL-1β, disrupting the balance between pro-inflammatory and anti-inflammatory factors (Victorelli et al., 2023; Jiménez-Loygorri and Boya, 2024; Marchi et al., 2023). Previous research had highlighted the role of IL-6 and other pro-inflammatory markers in aging and cognitive decline, supporting their potential as disease predictors (Weaver et al., 2002; Leonardo and Fregni, 2023; Lyra et al., 2021; Parks et al., 2020). Given these reports, we assessed inflammatory cytokines levels in the bloodstream and the results revealed that elevated plasma IL-6 levels were associated with *CF.*

This study has several limitations. Firstly, while *CF* should be defined as excluding dementia, our use of the PFP and MoCA scales as diagnostic tools, may have inadvertently included participants with early-stage dementia. Future follow-up studies are planned to more rigorously exclude cases of rapidly progressing dementia (Nasreddine et al., 2005; Malek-Ahmadi and Nikkhahmanesh, 2024). Additionally, there was a significant age difference between the two groups in our study. Although we re-analyzed the relationship between mitochondrial function and *CF*, it would be more rigorous to compare subjects within the same age range in both the non-*CF* and *CF* group. Next, other techniques for broader range of ROS assays is necessary to better understand the relationship between ROS and *CF*, such as spin trap compounds coupled with electron paramagnetic resonance spectroscopy, luminescence, mass spectrometry, and electrochemistry (Qu et al., 2020; Kornienko et al., 2018). Moreover, there was insufficient evidence to support the direct mechanistic roles of mitochondrial dysfunction, inflammation and *CF* in our study. Experimental studies should investigate mitochondria, metabolic and inflammatory alteration in the brain, such as metabolites levels in cerebrospinal fluid, PET brain glucose uptake, and magnetic resonance imaging. Additionally, the cross-sectional nature of this study limits our capacity to capture the temporal dynamics between mitochondrial function and *CF* progression. To address this, we will implement longitudinal follow-up assessments to better understand these relationships over time.

In summary, our findings highlight significant correlations between mitochondrial dysfunction and *CF*, suggesting that mitochondrial dysfunction in peripheral blood may serve as a potential phenotype of *CF.* These results underscore the need for further mechanistic studies to explore this relationship more comprehensively.

## Data Availability

The raw data supporting the conclusions of this article will be made available by the authors, without undue reservation.
